# A New Continuous Rotation IMU Alignment Algorithm Based on Stochastic Modeling for Cost Effective North-Finding Applications

**DOI:** 10.3390/s16122113

**Published:** 2016-12-13

**Authors:** Yun Li, Wenqi Wu, Qingan Jiang, Jinling Wang

**Affiliations:** 1 Department of Automatic Control, College of Mechatronics and Automation, National University of Defense Technology, Changsha 410073, China; liyun2009@nudt.edu.cn (Y.L.); jqa1987@nudt.edu.cn (Q.J.); 2 School of Civil and Environmental Engineering, UNSW Australia, Sydney 2052, NSW, Australia; jinling.wang@unsw.edu.au

**Keywords:** cost effective north-finding, stochastic modeling, Coriolis vibration gyroscopes, continuous rotation IMU alignment

## Abstract

Based on stochastic modeling of Coriolis vibration gyros by the Allan variance technique, this paper discusses Angle Random Walk (ARW), Rate Random Walk (RRW) and Markov process gyroscope noises which have significant impacts on the North-finding accuracy. A new continuous rotation alignment algorithm for a Coriolis vibration gyroscope Inertial Measurement Unit (IMU) is proposed in this paper, in which the extended observation equations are used for the Kalman filter to enhance the estimation of gyro drift errors, thus improving the north-finding accuracy. Theoretical and numerical comparisons between the proposed algorithm and the traditional ones are presented. The experimental results show that the new continuous rotation alignment algorithm using the extended observation equations in the Kalman filter is more efficient than the traditional two-position alignment method. Using Coriolis vibration gyros with bias instability of 0.1°/h, a north-finding accuracy of 0.1° (1*σ*) is achieved by the new continuous rotation alignment algorithm, compared with 0.6° (1*σ*) north-finding accuracy for the two-position alignment and 1° (1*σ*) for the fixed-position alignment.

## 1. Introduction

Cost effective north-finding technology is widely required for many applications. North-finding is sometimes based on Digital Magnetic Compasses (DMCs) [1]. However, DMCs is easily degraded by magnetic interference. Although Dynamically Tuned Gyros and Ring Laser Gyroscopes are suitable for precise north-finding, they are generally bulky and expensive [2,3]. In contrast, Coriolis vibration gyroscopes (e.g., a kind of cost effective medium precision Hemispherical Resonator Gyroscopes (HRGs) [4,5]) are generally compact and low-cost and suitable for a cost effective north-finding system. However, the drift errors of these gyroscopes are big problems, which limit the north-finding accuracy.

To improve the accuracy of the north-finding system using cost effective gyroscopes, several methods have been designed. Lee [6] proposed a multi-position alignment algorithm to increase the azimuth accuracy. For the same purpose, Yu [7] used analytic optimization of Strapdown Inertial Navigation System (SINS) multi-position alignment. Renkoski [8] and Sun [9] improved the accuracy of North-finding system through continuous rotation.

This paper focuses on Inertial Measurement Unit (IMU)-based north-finding systems using a Kalman filter for applications such as dynamic orientation and dead reckoning. Stochastic modeling for a Coriolis vibration gyroscope is obtained using the Allan variance technique. It is shown that the Rate Random Walk (RRW) and Markov noises are the main errors which limit the north-finding accuracy. A new continuous rotation IMU alignment algorithm is therefore proposed using extended observation equations in the Kalman filter to solve this problem. Experimental results as well as theoretical analysis are also presented.

This paper is organized as follows: Section 2 analyses the random error model of a Coriolis vibration gyroscope using the Allan variance technique. The north-finding errors due to the main parts of the gyro drift error are presented. Section 3 presents three different IMU based north-finding algorithms or three different error compensation approaches: two-position alignment, continuous rotation alignment, and a new continuous rotation alignment algorithm with extended observation equations for a Kalman filter. Section 4 presents theoretical and simulation analyses of the performances of the methods mentioned above. Section 5 reports north-finding experimental results and comparisons. The Allan variance analysis results for the equivalent east gyro are presented for the interpretation of effectiveness of the gyro drift error compensation approaches. Section 6 concludes the paper. The appendices show detailed theoretical proofs.

## 2. Error Model for a Coriolis Vibration Gyroscope

IMU errors can be classified into two types: deterministic errors and random errors. Major deterministic error sources including constant bias, scale factor errors and misalignment can be removed by calibration and compensation [10]. The random constant bias (turn to turn bias) and random noises are the main error sources in the North-finding system. Therefore, we focus on the stochastic modeling for a Coriolis vibration gyroscope.

### 2.1. Error Model Based on Allan Variance Analysis

Traditionally, random constant bias, ARW (Angle Random Walk), RRW and Markov process are used to develop stochastic error model for gyros. The error model of a gyroscope can be expressed as follows [11,12]:

(1) $$\varepsilon =\varepsilon_{b}+\varepsilon_{m}+w_{a}+\varepsilon_{r}$$

where ε is the stochastic drift error of the gyroscope measurements, $\varepsilon_{b}$ is the random constant bias with the variance of $\sigma_{b}^{2}$, $\varepsilon_{m}$ is the Markov process, $w_{a}$ is the ARW, $\varepsilon_{r}$ is the RRW.

The random bias can be described as an unpredictable random quantity with a constant value, that is:

(2) $${\dot{\varepsilon }}_{b}=0$$

(3) $$w_{a}\in N(0,\sigma_{a}^{2})$$

where $\sigma_{a}^{2}$ is the variance of $w_{a}$.

The Markov noise is the low-frequency component in the error sources. Usually, the noise is modeled as a First order Gauss-Markov process [11]:

(4) $${\dot{\varepsilon }}_{m}=-\frac{1}{\tau }\varepsilon_{m}+w_{m},\;w_{m}\in N(0,\sigma_{m}^{2})$$

where τ is the process time constant, $w_{m}$ is the zero-mean Gaussian white noise, $\sigma_{m}^{2}$ is the variance of $w_{m}$:

(5) $${\dot{\varepsilon }}_{r}=w_{r},\;w_{r}\in N(0,\sigma_{r}^{2})$$

where $\sigma_{r}^{2}$ is the variance of $w_{r}$.

**Figure 1:**
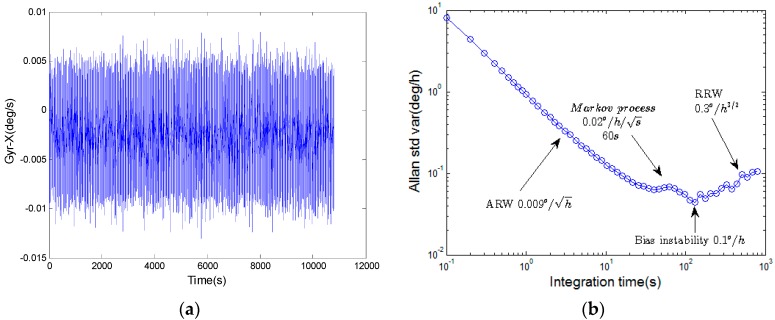
(**a**) Measurements of a HRG in 10Hz; (**b**). Allan variance of the HRG.

In Equation (1), the characteristics of the stochastic errors are usually estimated by an optimal estimation algorithm, such as a Kalman filter [13]. The parameters of the stochastic error model are necessary for a Kalman filter algorithm. Hence, there is a need to determine the parameters of the error model using Allan variance analysis. The sampling data of a HRG in 3 h is present in Figure 1a. The Allan variance results of the HRG are presented in Figure 1b. The sampling frequency is 10 Hz.

The parameters of the error models for the Coriolis vibration gyroscopes in an IMU based north-finding system are given in Table 1.

Consider the error models in Figure 1, the major parts of the gyroscope errors are ARW, Markov process, bias instability and RRW, which indicates that the error model in Equation (1) is sufficient to characterize the gyroscope. The parameters of the models show that the primary error source for the gyroscope are Markov noise and RRW.

### 2.2. Propagation of Gyroscope Errors in a North-Finding System

The drift error of the equivalent east gyroscope $\varepsilon_{E}$ in an IMU based north-finding system propagates to the azimuth misalignment $\phi_{D}$, which can be expressed as follows [14]:

(6) $$\phi_{D}=\frac{\varepsilon_{E}}{\Omega \cos L}$$

where Ω is the earth rotation rate, L is the local latitude.

Similar to Equation (1), $\varepsilon_{E}$ can be expressed as follows:

(7) $$\varepsilon_{E}=\varepsilon_{bE}+w_{aE}+\varepsilon_{rE}+\varepsilon_{mE}$$

where the random constant bias $\varepsilon_{bE}$, the ARW $w_{aE}$, the RRW $\varepsilon_{rE}$ and the Markov process $\varepsilon_{mE}$ correspond to $\varepsilon_{b}$, $w_{a}$, $\varepsilon_{r}$ and $\varepsilon_{m}$ in Equation (1).

The RMS (Root Mean Square) of azimuth misalignment $\sigma_{\phi_{Db}}$, $\sigma_{\phi_{Da}}$, $\sigma_{\phi_{Dr}}$ and $\sigma_{\phi_{Dm}}$ due to $\varepsilon_{bE}$, $w_{aE}$, $\varepsilon_{rE}$ and $\varepsilon_{mE}$ can be expressed as [15,16]:

(8) $$\sigma_{\phi_{Db}}=\frac{\sigma_{bE}}{\Omega \cos L}$$

(9) $$\sigma_{\phi_{Da}}=\frac{\sigma_{aE}}{\sqrt{t_{n}}\Omega \cos L}$$

(10) $$\sigma_{\phi_{Dr}}=\frac{\sigma_{rE}\sqrt{t_{n}}}{\sqrt{3}\Omega \cos L}$$

(11) $$\sigma_{\phi_{Dm}}=\frac{\sqrt{\frac{1}{2}\tau^{2}\sigma_{mE}^{2}(2t-\tau e^{-\frac{2t}{\tau }}+4\tau e^{-\frac{t}{\tau }}-3\tau )+P_{\varepsilon_{mE}}(0)\tau (1-2e^{-\frac{1}{\tau }t}+e^{-\frac{2}{\tau }t})}}{t_{n}\Omega \cos L}$$

where $t_{n}$ is the alignment time. $\sigma_{bE}^{2}$, $\sigma_{aE}^{2}$, $\sigma_{rE}^{2}$ and $\sigma_{mE}^{2}$ are the variances corresponding to $\varepsilon_{bE}$, $w_{aE}$, $w_{rE}$ and $w_{mE}$ in the error model of the equivalent east gyroscope. $P_{\varepsilon_{mE}}(0)$ is the variance of the initial value of Markov process. The proofs of Equations (8)–(11) are shown in Appendix A.

It should be explained that the initial value of RRW noise can be regarded as part of a constant bias. Thus the RRW starts from zero.

Assuming the alignment time $t_{n}$ is 10 min, the local latitude is 28.22° N, the RMS values of the azimuth misalignment can be obtained from Equations (8)–(11). The azimuth misalignment due to the equivalent east gyroscope errors are shown in Table 2.

Although the azimuth misalignment are most affected by the bias instability, the random constant bias can be easily eliminated through north-finding algorithms (such as two-position alignment [6] and continuous rotation alignment [9]). And compared with RRW and Markov noise, the azimuth misalignment due to ARW is slim. RRW and Markov process are the main error source in a north-finding system.

## 3. Error Compensation Approach for IMU Based North-Finding System

### 3.1. System Error Model for IMU Based North-Finding

A local level NED (North-East-Down) frame is used as the navigation frame. The common SINS error equations in the navigation frame can be expressed as follows [14]:

(12) $${\dot{\phi }}^{n}=-(\omega_{en}^{n}+\omega_{ie}^{n})\times \phi^{n}-C_{b}^{n}\delta \omega_{ib}^{b}$$

(13) $$\delta {\dot{v}}^{n}=f^{n}\times \phi^{n}-(\omega_{en}^{n}+2\omega_{ie}^{n})\times \delta v^{n}+C_{b}^{n}\delta f^{b}$$

where $\phi^{n}$ is the attitude error, $\phi^{n}={\left[\begin{array}{ccc} \phi_{N} & \phi_{E} & \phi_{D} \end{array}\right]}^{T}$, N, E and D represent north, east and down in navigation frame respectively; $\delta v^{n}$ is the velocity error, $\delta v^{n}={\left[\begin{array}{ccc} \delta v_{N} & \delta v_{E} & \delta v_{D} \end{array}\right]}^{T}$. $\phi^{n}$ can be estimated by the observation of $\delta v^{n}$ in an alignment process. $f^{n}$ is the measurement of specific force in frame *n*, $C_{b}^{n}$ is the coordinate transformation matrix from the IMU frame *b* to the navigation frame *n*, $\omega_{en}^{n}$ is the turn rate of the navigation frame to the earth frame in the frame *n*, $\omega_{ie}^{n}$ is the turn rate of the earth frame to the inertial frame in the frame *n*, $\delta \omega_{ib}^{b}$ is the error of the gyroscope measurements, $\delta f^{b}$ is the error of the specific force measurements.

In the North finding scenario discussed here, since the IMU is stationary on the Earth:

(14) $$\omega_{en}^{n}\;=\;0$$

The SINS error model for alignment or IMU based north-finding can be written as:

(15) $$\dot{x}(t)=\left[\begin{array}{cc} F_{1} & F_{2}\\ 0_{8\times 5} & \Gamma \end{array}\right]\dot{x}(t)+\left[\begin{array}{cc} G_{1} & 0_{5\times 3}\\ 0_{8\times 5} & \begin{array}{c} 0_{5\times 3}\\ I_{3\times 3} \end{array} \end{array}\right]w(t)$$

where:

(16) $$\begin{array}{l} x(t)=[\begin{array}{ccccc} \delta v_{N} & \delta v_{E} & \phi_{N} & \phi_{E} & \begin{array}{cc} \phi_{D} & \begin{array}{cc} \nabla_{accx} & \nabla_{accy} \end{array} \end{array} \end{array}\\ {\begin{array}{cccccc} \left(\varepsilon_{bx}+\varepsilon_{rx}\right) & \left(\varepsilon_{by}+\varepsilon_{ry}\right) & \left(\varepsilon_{bz}+\varepsilon_{rz}\right) & \varepsilon_{mx} & \varepsilon_{my} & \varepsilon_{mz} \end{array}]}^{T} \end{array}$$

$\nabla_{accx}$ and $\nabla_{accy}$ are the bias error states of the accelerometers, $\varepsilon_{bx}$, $\varepsilon_{by}$ and $\varepsilon_{bz}$ are the random constant bias error states of the gyroscopes, $\varepsilon_{rx}$, $\varepsilon_{ry}$ and $\varepsilon_{rz}$ are the rate random walk of the gyroscopes, $\varepsilon_{mx}$, $\varepsilon_{my}$ and $\varepsilon_{mz}$ are the error states for the Markov process of the gyroscopes.

For the filter noise vector:

(17) $$w(t)\;=\;{\left[\begin{array}{ccccc} w_{accx} & w_{accy} & w_{ax} & w_{ay} & \begin{array}{ccccccc} w_{az} & w_{rx} & w_{ry} & w_{rz} & w_{mx} & w_{my} & w_{mz} \end{array} \end{array}\right]}^{T}$$

where $w_{accx}$ and $w_{accy}$ are the white noise of the accelerometer x and the accelerometer y, respectively. That is:

(18) $$w_{accx},w_{accy}\in N(0,\sigma_{acc}^{2})$$

where $\sigma_{acc}^{2}$ is the variance of the white noise $w_{accx}$ and $w_{accy}$.

$w_{ax}$, $w_{ay}$ and $w_{az}$ are the angular random walk of the gyroscope *x*, the gyroscope *y* and the gyroscope *z*, $w_{mx}$, $w_{my}$ and $w_{mz}$ are the driving noise in the Markov process of the gyroscope *x*, the gyroscope *y* and the gyroscope *z*.

(19) $$F_{1}=\left[\begin{array}{ccccc} 0 & -2\Omega \sin L & 0 & g & 0\\ 2\Omega \sin L & 0 & -g & 0 & 0\\ 0 & 0 & 0 & -\Omega \sin L & 0\\ 0 & 0 & \Omega \sin L & 0 & \Omega \cos L\\ 0 & 0 & 0 & -\Omega \cos L & 0 \end{array}\right]$$

where g is the local gravity.

The matrix $F_{2}$ is defined as follows:

(20) $$F_{2}=\left[\begin{array}{ccc} -{C_{b}^{n}}_{2\times 2} & 0_{2\times 3} & 0_{2\times 3}\\ 0_{3\times 2} & -C_{b}^{n} & -C_{b}^{n} \end{array}\right]$$

where ${C_{b}^{n}}_{2\times 2}$ is defined as:

(21) $${C_{b}^{n}}_{2\times 2}=\left[\begin{array}{ccc} 1 & 0 & 0\\ 0 & 1 & 0 \end{array}\right]C_{b}^{n}{\left[\begin{array}{ccc} 1 & 0 & 0\\ 0 & 1 & 0 \end{array}\right]}^{T}$$

The matrices $G_{1}$ and Γ are defined as follows:

(22) $$G_{1}=\left[\begin{array}{cc} {C_{b}^{n}}_{2\times 2} & 0_{2\times 3}\\ 0_{3\times 2} & -C_{b}^{n} \end{array}\right],\;\Gamma =\left[\begin{array}{cc} 0_{5\times 5} & 0_{5\times 3}\\ 0_{3\times 5} & -\frac{1}{\tau }I_{3\times 3} \end{array}\right]$$

where τ is the Markov time constant of the gyroscope.

As shown in the analysis above, based on the condition that the system is stationary on the earth, the horizontal velocity errors are used as observation states. Thus, the observation model can be written as:

(23) $$z(t)=\left[\begin{array}{c} {\tilde{v}}_{N}\\ {\tilde{v}}_{E} \end{array}\right]=Hx(t)+\upsilon (t)\;H=\left[\begin{array}{cc} I_{2\times 2} & 0_{2\times 11} \end{array}\right]$$

where $\upsilon (t)={\left[\begin{array}{cc} \upsilon_{v_{N}} & \upsilon_{v_{E}} \end{array}\right]}^{T}$ is the observation noise vector. ${\tilde{v}}_{N}$ and ${\tilde{v}}_{E}$ represent north and east components of the estimated velocity, respectively.

### 3.2. Traditional Two-Position Gyrocompassing

**Figure 2:**
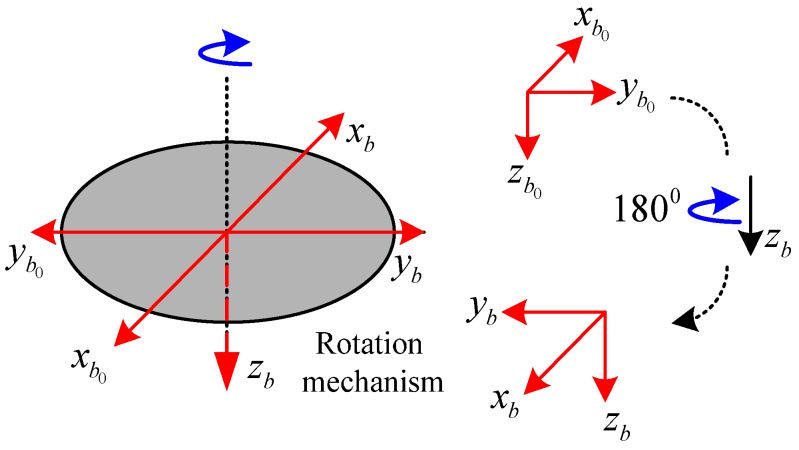
Schematic diagram of two-position alignment.

Two-position alignment is demonstrated in Figure 2 [6].

As shown in Figure 2, the axis $x_{b}$ and $y_{b}$ of the IMU frame lie on the turntable plane, the axis $z_{b}$ coincides with the rotation axis. We define the $b_{0}$ frame when $x_{b}$ coincides with the turntable null indicator:

(24) $$C_{n}^{b}=C_{b_{0}}^{b}C_{n}^{b_{0}}$$

where $C_{n}^{b_{0}}$ is the coordinate transformation matrix from the frame n to the frame $b_{0}$.

$C_{b_{0}}^{b}$ can be written as:

(25) $$C_{b_{0}}^{b}(t)=\{\begin{array}{c} \left[\begin{array}{ccc} 1 & 0 & 0\\ 0 & 1 & 0\\ 0 & 0 & 1 \end{array}\right]\begin{array}{c} t<t_{1} \end{array}\\ \left[\begin{array}{ccc} -1 & 0 & 0\\ 0 & -1 & 0\\ 0 & 0 & 1 \end{array}\right]\begin{array}{c} t>t_{2} \end{array} \end{array}$$

where $\left[t_{1},t_{2}\right]$ is the short time period when the IMU changes the angular position through the turntable rotation.

### 3.3. Continuous Rotation Gyrocompassing

As an alternative to the two-position alignment, continuous rotation is another efficient method to reduce the alignment errors.

In contrast to the two-position alignment, the coordinate transformation matrix $C_{b}^{n}$ is varying as $C_{b}^{b_{0}}$ changes by continuous rotation, that is:

(26) $$C_{b}^{b_{0}}=\left[\begin{array}{ccc} \cos(\omega_{0}t) & \sin(\omega_{0}t) & 0\\ -\sin(\omega_{0}t) & \cos(\omega_{0}t) & 0\\ 0 & 0 & 1 \end{array}\right]\;\omega_{0}T=2\pi$$

where $\omega_{0}$ is the rotation rate of the turntable. *T* is the rotation cycle.

Except for the coordinate transformation matrix $C_{b}^{n}$ and $C_{b}^{b_{0}}$, the error model and the observation equation between the continuous rotation gyrocompassing are the same as that of the two-position gyrocompassing.

### 3.4. A New Continuous Rotation North-Finding Method Based on an Extended Observation Model

Although the constant random biases of gyroscopes are mostly eliminated by the above compensation approaches, the noise of the gyroscopes will also still affect the efficiency of the Kalman filter. For Coriolis vibration gyroscopes, the noise level is high. It is difficult to estimate the drift errors of the gyroscopes exactly. The accuracy of the North-finding system is limited. To solve the problem, we present an extended observation model for the continuous rotation alignment.

After each 360° turn, the integration of the measurements of the gyroscopes can be written as:

(27) $$\int_{t}^{t+T}{\tilde{\omega }}_{ib}^{b}dt=\int_{t}^{t+T}\omega_{ib}^{b}dt+\int_{t}^{t+T}\left[\begin{array}{c} (\varepsilon_{bx}+\varepsilon_{rx})+\varepsilon_{mx}\\ (\varepsilon_{by}+\varepsilon_{ry})+\varepsilon_{my}\\ (\varepsilon_{bz}+\varepsilon_{rz})+\varepsilon_{mz} \end{array}\right]dt\approx \int_{t}^{t+T}\left(\omega_{eb}^{b}+C_{b_{0}}^{b}C_{n}^{b_{0}}\omega_{ie}^{n}\right)dt+T\left[\begin{array}{c} (\varepsilon_{bx}+\varepsilon_{rx})+\varepsilon_{mx}\\ (\varepsilon_{by}+\varepsilon_{ry})+\varepsilon_{my}\\ (\varepsilon_{bz}+\varepsilon_{rz})+\varepsilon_{mz} \end{array}\right]$$

While the integration of the estimated measurements of the gyroscopes can be written as:

(28) $$\begin{array}{l} \int_{t}^{t+T}{\hat{\omega }}_{ib}^{b}dt=\int_{t}^{t+T}\left(\omega_{eb}^{b}+{\hat{\omega }}_{ie}^{b}\right)dt\\ =\int_{t}^{t+T}\omega_{eb}^{b}dt+\int_{t}^{t+T}{\tilde{C}}_{n}^{b}{\hat{\omega }}_{ie}^{n}dt\\ =\int_{t}^{t+T}\omega_{eb}^{b}dt+\int_{t}^{t+T}\{C_{b_{0}}^{b}C_{n}^{b_{0}}[I+\phi^{n}\times ]\omega_{ie}^{n}+C_{b_{0}}^{b}C_{n}^{b_{0}}\delta \omega_{ie}^{n}\}dt\\ =\int_{t}^{t+T}\left(\omega_{eb}^{b}+C_{b_{0}}^{b}C_{n}^{b_{0}}\omega_{ie}^{n}\right)dt+\int_{t}^{t+T}\{C_{b_{0}}^{b}C_{n}^{b_{0}}[-\omega_{ie}^{n}\times ]\phi^{n}+C_{b_{0}}^{b}C_{n}^{b_{0}}\delta \omega_{ie}^{n}\}dt \end{array}$$

(29) $$\begin{array}{c} \;\omega_{ie}^{n}=\left[\begin{array}{c} \Omega \cos L\\ 0\\ -\Omega \sin L \end{array}\right]\;\delta \omega_{ie}^{n}=\left[\begin{array}{c} -\delta L\Omega \sin L\\ 0\\ -\delta L\Omega \cos L \end{array}\right]\;\\ C_{n}^{b_{0}}=\left[\begin{array}{ccc} \cos\theta_{0}\cos\varphi_{0} & \cos\theta_{0}\sin\varphi_{0} & -\sin\theta_{0}\\ -\cos\gamma_{0}\sin\varphi_{0}+\sin\gamma_{0}\sin\theta_{0}\cos\varphi_{0} & \cos\theta_{0}\cos\varphi_{0}+\sin\gamma_{0}\sin\theta_{0}\sin\varphi_{0} & \sin\gamma_{0}\cos\theta_{0}\\ \cos\gamma_{0}\sin\theta_{0}\cos\varphi_{0}+\sin\gamma_{0}\sin\varphi_{0} & -\sin\gamma_{0}\cos\varphi_{0}+\cos\gamma_{0}\sin\theta_{0}\sin\varphi_{0} & \cos\gamma_{0}\cos\theta_{0} \end{array}\right] \end{array}$$

where $\int_{t}^{t+T}{\tilde{\omega }}_{ib}^{b}dt$ represents the integration of the gyroscope measurements in a rotation cycle of the turntable, ${\hat{\omega }}_{ib}^{b}$ represents the estimated measurements of the gyroscopes in the *b*-frame, $\omega_{ie}^{n}$ represents the earth rotation rate in the *n*-frame. $\varphi_{0}$, $\theta_{0}$ and $\gamma_{0}$ are the Euler angles of the $b_{0}$-frame relative to the *n*-frame. ${\tilde{C}}_{n}^{b}$ is the estimated coordinate transformation matrix with attitude errors.

Considering $\phi_{N}$ and $\phi_{E}$ are very small after coarse alignment:

(30) $$\phi^{n}=\left[\begin{array}{c} \phi_{N}\\ \phi_{E}\\ \phi_{D} \end{array}\right]\approx \left[\begin{array}{c} 0\\ 0\\ \phi_{D} \end{array}\right]$$

From Equations (29) and (30)

(31) $$\int_{t}^{t+T}C_{b_{0}}^{b}C_{n}^{b_{0}}\omega_{ie}^{n}dt=\Omega T\left[\begin{array}{c} 0\\ 0\\ \cos L(\cos\gamma_{0}\sin\theta_{0}\cos\varphi_{0}+\sin\gamma_{0}\sin\varphi_{0})-\sin L\cos\gamma_{0}\cos\theta_{0} \end{array}\right]$$

(32) $$\begin{array}{l} \int_{t}^{t+T}\delta {\hat{\omega }}_{ib}^{b}dt\\ =\int_{t}^{t+T}\{C_{b_{0}}^{b}C_{n}^{b_{0}}[-\omega_{ie}^{n}\times ]\phi^{n}+C_{b_{0}}^{b}C_{n}^{b_{0}}\delta \omega_{ie}^{n}\}dt\\ =\phi_{D}\Omega T\cos L\left[\begin{array}{c} 0\\ 0\\ \left(\begin{array}{l} -\sin\gamma_{0}\cos\varphi_{0}+\\ \cos\gamma_{0}\sin\theta_{0}\sin\varphi_{0} \end{array}\right) \end{array}\right]+\delta L\Omega T\left[\begin{array}{c} 0\\ 0\\ \left(\begin{array}{l} -\sin L(\cos\gamma_{0}\sin\theta_{0}\cos\varphi_{0}+\sin\gamma_{0}\sin\varphi_{0})-\\ \cos L\cos\gamma_{0}\cos\theta_{0} \end{array}\right) \end{array}\right] \end{array}$$

Under static conditions, we have:

(33) $$\int_{t}^{t+T}\omega_{eb}^{b}dt=\left[\begin{array}{c} 0\\ 0\\ 2\pi \end{array}\right]$$

Substituting Equations (28), (31)–(33) into Equation (27) gives:

(34) $$\begin{array}{l} T\left[\begin{array}{c} (\varepsilon_{bx}+\varepsilon_{rx})+\varepsilon_{mx}\\ (\varepsilon_{by}+\varepsilon_{ry})+\varepsilon_{my}\\ (\varepsilon_{bz}+\varepsilon_{rz})+\varepsilon_{mz} \end{array}\right]-\phi_{D}\Omega T\cos L\left[\begin{array}{c} 0\\ 0\\ \left(\begin{array}{l} -\sin\gamma_{0}\cos\varphi_{0}+\\ \cos\gamma_{0}\sin\theta_{0}\sin\varphi_{0} \end{array}\right) \end{array}\right]-\delta L\Omega T\left[\begin{array}{c} 0\\ 0\\ \left(\begin{array}{l} -\sin L\cos\gamma_{0}\sin\theta_{0}\cos\varphi_{0}\\ -\sin L\sin\gamma_{0}\sin\varphi_{0}\\ -\cos L\cos\gamma_{0}\cos\theta_{0} \end{array}\right) \end{array}\right]\\ =\int_{t}^{t+T}{\tilde{\omega }}_{ib}^{b}dt-\int_{t}^{t+T}{\hat{\omega }}_{ib}^{b}dt\\ =\int_{t}^{t+T}{\tilde{\omega }}_{ib}^{b}dt-\Omega T\left[\begin{array}{c} 0\\ 0\\ (\cos\gamma_{0}\sin\theta_{0}\cos{\hat{\varphi }}_{0}+\sin\gamma_{0}\sin{\hat{\varphi }}_{0})\cos\hat{L}-\cos\gamma_{0}\cos\theta_{0}\sin\hat{L} \end{array}\right]-\left[\begin{array}{c} 0\\ 0\\ 2\pi \end{array}\right] \end{array}$$

When there is latitude error and heading error, the estimated measurements of the gyroscopes are inaccurate. After each 360° turn of the turntable, the equivalent east gyroscope error caused by these errors can be calculated as follows:

(35) $$\delta \omega_{ibE}^{n}=\frac{1}{T}\left[\begin{array}{ccc} 0 & 1 & 0 \end{array}\right]\int_{t}^{t+T}C_{b_{0}}^{n}C_{b}^{b_{0}}\delta {\hat{\omega }}_{ib}^{b}dt$$

The equivalent east gyroscope error caused by heading error and latitude error is shown in Equations (36) and (37) respectively:

(36) $$\delta \omega_{ibE,\phi_{D}}^{n}={\left(-\sin\gamma_{0}\cos\varphi_{0}+\cos\gamma_{0}\sin\theta_{0}\sin\varphi_{0}\right)}^{2}\phi_{D}\Omega \cos L$$

(37) $$\begin{array}{cc} \delta \omega_{ibE,\delta L}^{n} & =(\sin\gamma_{0}\cos\varphi_{0}-\cos\gamma_{0}\sin\theta_{0}\sin\varphi_{0})[\cos\gamma_{0}\sin\theta_{0}\cos\varphi_{0}\tan L\\ & +\sin\gamma_{0}\sin\varphi_{0}\tan L+\cos\gamma_{0}\cos\theta_{0}]\delta L\Omega \cos L \end{array}$$

where $\delta \omega_{ibE,\phi_{D}}^{n}$ is the equivalent east gyroscope error caused by heading error, $\delta \omega_{ibE,\delta L}^{n}$ is the equivalent east gyroscope error caused by latitude error δL.

Assuming that:

(38) $$\gamma_{0}=\theta_{0}=5{}^{\circ}$$

Equations (36) and (37) can be written as:

(39) $$\delta \omega_{ibE,\phi_{D}}^{n}\approx 0.01\phi_{D}\Omega \cos L,\;\delta \omega_{ibE,\delta L}^{n}\approx 0.1\delta L\Omega \cos L$$

In general, the initial heading error is less than 5° ($\phi_{D}(0)<5{}^{\circ}$) and the latitude error is less than 0.1° (δL<0.1°). Considering Equation (39), the equivalent azimuth error caused by initial heading error and latitude error can be ignored when tilt is smaller than 5°.

The additional observation can be obtained using the integration measurements of the gyroscopes in each 360° turn of the turntable.

The observation model can be written as:

(40) z(t)=Hx(t)+υ(t)

(41) $$\begin{array}{l} z(t)=\{\begin{array}{c} \left[\begin{array}{c} {\tilde{v}}_{N}\\ {\tilde{v}}_{E} \end{array}\right]\begin{array}{c} when\;2(k-1)\pi <\omega_{0}t<2k\pi \end{array}\\ \left[\begin{array}{c} {\tilde{v}}_{N}\\ {\tilde{v}}_{E}\\ \int_{t}^{t+T}{\tilde{\omega }}_{ib}^{b}dt-\int_{t}^{t+T}{\hat{\omega }}_{ib}^{b}dt \end{array}\right]\begin{array}{c} when\;\omega_{0}t=2k\pi \end{array} \end{array}\\ \;k=1,2,3,\ldots \end{array}$$

(42) $$\begin{array}{l} H=\{\begin{array}{c} \left[\begin{array}{ccc} I_{2\times 2} & 0_{2\times 3} & 0_{2\times 8} \end{array}\right]\begin{array}{c} when\;2(k-1)\pi <\omega_{0}t<2k\pi \end{array}\\ \left[\begin{array}{ccccc} I_{2\times 2} & 0_{2\times 3} & 0_{2\times 2} & 0_{2\times 3} & 0_{2\times 3}\\ 0_{3\times 2} & D & 0_{3\times 2} & TI_{3\times 3} & TI_{3\times 3} \end{array}\right]\begin{array}{c} when\;\omega_{0}t=2k\pi \end{array} \end{array}\\ \;k=1,2,3,\ldots \\ D=\left[\begin{array}{ccc} 0 & 0 & 0\\ 0 & 0 & 0\\ 0 & 0 & (-\sin\gamma_{0}\cos\varphi_{0}+\cos\gamma_{0}\sin\theta_{0}\sin\varphi_{0})\Omega T\cos L \end{array}\right] \end{array}$$

(43) $$\begin{array}{l} \upsilon (t)=\{\begin{array}{c} {\left[\begin{array}{cc} \upsilon_{v_{N}} & \upsilon_{v_{E}} \end{array}\right]}^{T}\begin{array}{c} when\;2(k-1)\pi <\omega_{0}t<2k\pi \end{array}\\ \begin{array}{cc} {\left[\begin{array}{ccccc} \upsilon_{v_{N}} & \upsilon_{v_{E}} & \upsilon_{\omega_{x}} & \upsilon_{\omega_{y}} & \upsilon_{\omega_{z}} \end{array}\right]}^{T} & when\;\omega_{0}t=2k\pi \end{array} \end{array}\\ \;k=1,2,3,\ldots \end{array}$$

where $\upsilon_{\omega_{x}}$, $\upsilon_{\omega_{y}}$ and $\upsilon_{\omega_{z}}$ are the observation noise corresponding to $\int_{t}^{t+T}{\tilde{\omega }}_{ib}^{b}dt-\int_{t}^{t+T}{\hat{\omega }}_{ib}^{b}dt$. 

## 4. Comparisons of the Kalman Filter Convergence Rapidity and North-Finding Accuracy

Comparisons of the Kalman filter convergence rapidity and the north-finding accuracy between the proposed algorithms and the traditional alignment methods can be made with the covariance matrix for the estimated states in the Kalman filter.

For the piecewise constant time varying system the covariance matrix of the estimated states P can be obtained by calculating the discrete Riccati matrix equation [7]:

(44) $$\begin{array}{l} P^{-1}(k)={\left[\Phi^{T}(k,k-1)P(k-1)\Phi (k,k-1)+G^{T}QG\right]}^{-1}\\ \;+H^{T}R^{-1}H\;k=1,2,3\ldots ,n \end{array}$$

which is based on the continuous system error model and observation equations (Equations (15)–(43)) as follows:

(45) $$\begin{array}{l} \Phi (k,k-1)\approx e^{A(t_{k-1})T_{s}}\approx I+A(t_{k-1})T_{s}\\ G(k,k-1)\approx B(t_{k-1})\\ Q=qT_{s}\\ q=E\{w^{T}(t)w(t)\}\\ R=E\{\upsilon^{T}(t)\upsilon (t)\} \end{array}$$

where $T_{s}=0.04\;s$ is the sampling time. 

In this study, an initial covariance matrix $P_{1}(0)$, spectral density matrix Q of system noise and measurement noise covariance matrix R are given as follows:

(46) $$\begin{array}{l} P_{1}(0)=diag\{{\left(0.1\;m/s\right)}^{2},{\left(0.1\;m/s\right)}^{2},{\left(1{}^{\circ}\right)}^{2},{\left(1{}^{\circ}\right)}^{2},{\left(1{}^{\circ}\right)}^{2},\\ {\left(10^{-4}g\right)}^{2},{\left(10^{-4}g\right)}^{2},{\left(0.2{}^{\circ}/h\right)}^{2},{\left(0.2{}^{\circ}/h\right)}^{2},{\left(0.2{}^{\circ}/h\right)}^{2},{\left(0.2{}^{\circ}/h\right)}^{2},{\left(0.2{}^{\circ}/h\right)}^{2},{\left(0.2{}^{\circ}/h\right)}^{2}\}\\ Q=diag\{{\left(2\times 10^{-5}\;m/s\right)}^{2},{\left(2\times 10^{-5}\;m/s\right)}^{2},{\left(0.12^{\prime \prime }\right)}^{2},{\left(0.12^{\prime \prime }\right)}^{2},{\left(0.12^{\prime \prime }\right)}^{2},\\ {\left(0.0001{}^{\circ}/h\right)}^{2},{\left(0.0001{}^{\circ}/h\right)}^{2},{\left(0.0001{}^{\circ}/h\right)}^{2},{\left(0.004{}^{\circ}/h\right)}^{2},{\left(0.004{}^{\circ}/h\right)}^{2},{\left(0.004{}^{\circ}/h\right)}^{2}\}\\ R=diag\{{\left(0.01\;m/s\right)}^{2},{\left(0.01\;m/s\right)}^{2}\} \end{array}$$

When using the continuous rotation method based on the extended observation model, measurement noise covariance matrix R is expressed as follows:

(47) $$\begin{array}{l} R=\{\begin{array}{c} \begin{array}{c} diag\{{\left(0.01\;m/s\right)}^{2},{\left(0.01\;m/s\right)}^{2}\}\begin{array}{c} when\;2(k-1)\pi <\omega_{0}t<2k\pi \end{array} \end{array}\\ \begin{array}{cc} diag\{{\left(0.01\;m/s\right)}^{2},{\left(0.01\;m/s\right)}^{2},{\left(4^{\prime \prime }\right)}^{2},{\left(4^{\prime \prime }\right)}^{2},{\left(4^{\prime \prime }\right)}^{2}\} & when\;\omega_{0}t=2k\pi \end{array} \end{array}\\ \;k=1,2,3,\ldots \end{array}$$

The rotation rate of the turntable is $\omega_{0}=10{}^{\circ}/s$. The number of iterations performed for calculating P using Equation (44) is 15,000 which is equivalent to 600 s. For two-position alignment, the IMU changes position at 300 s. Since the heading error $\phi_{D}$ is the most crucial error state in the north-finding system, we focus on the RMS value of $\phi_{D}$.

**Figure 3:**
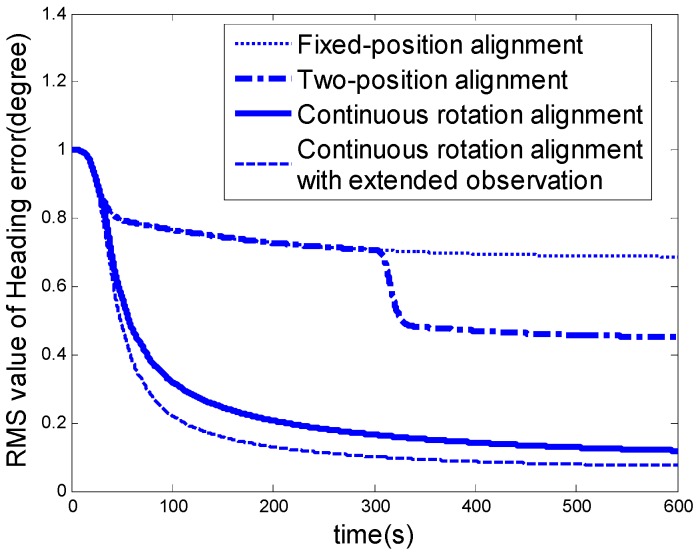
Kalman filter convergence rapidity and accuracy comparison of the four north-finding approaches.

Figure 3 shows the RMS values of the heading error in the north-finding process. Obviously, the new continuous rotation alignment with the extended observation is more efficient than the existing north-finding algorithms.

In order to analyze the gyroscope error compensation effect of the new continuous rotation alignment approach, we use Allan variance technique to compare the compensated data with the uncompensated data of the equivalent east gyroscope, which determines the north-finding accuracy in a north-finding system.

The uncompensated equivalent east gyroscope data, denoted as ${\tilde{\omega }}_{ibE}^{n}$ is the measurement of the equivalent east gyroscope in the *n* frame, when the turntable is not rotating, that is:

(48) $${\tilde{\omega }}_{ibE}^{n}=[\begin{array}{ccc} 0 & 1 & 0 \end{array}]C_{b_{0}}^{n}C_{b}^{b_{0}}{\tilde{\omega }}_{ib}^{b}\;\begin{array}{c} C_{b}^{b_{0}}=I \end{array}$$

The compensated equivalent east gyroscope data, denoted as ${\hat{\omega }}_{ibE}^{n}$ is the measurement of the equivalent east gyroscope in the *n* frame, when the turntable is rotating. The compensated data is obtained after the Kalman filter has converged. The drift error of the gyroscope has been estimated and compensated by the Kalman filter. That is:

(49) $${\hat{\omega }}_{ibE}^{n}=[\begin{array}{ccc} 0 & 1 & 0 \end{array}]C_{b_{0}}^{n}C_{b}^{b_{0}}\left({\tilde{\omega }}_{ib}^{b}-\left[\begin{array}{c} {\hat{\varepsilon }}_{bx}+{\hat{\varepsilon }}_{rx}\\ {\hat{\varepsilon }}_{by}+{\hat{\varepsilon }}_{ry}\\ {\hat{\varepsilon }}_{bz}+{\hat{\varepsilon }}_{rz} \end{array}\right]-\left[\begin{array}{c} {\hat{\varepsilon }}_{mx}\\ {\hat{\varepsilon }}_{my}\\ {\hat{\varepsilon }}_{mz} \end{array}\right]\right)$$

**Figure 4:**
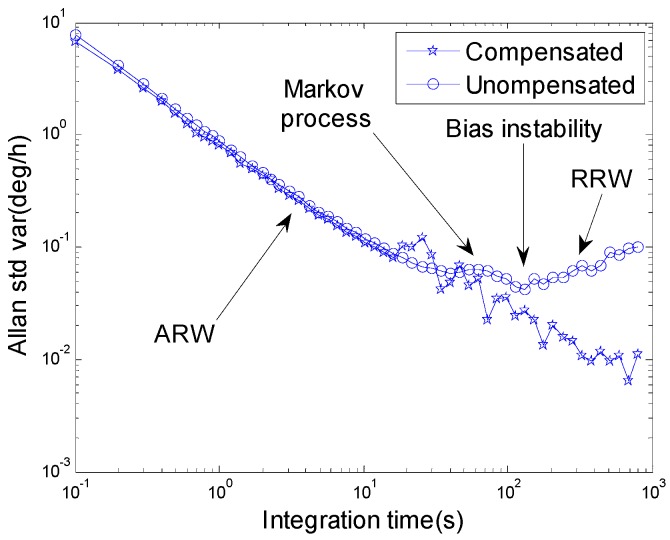
Allan variance of compensated and uncompensated data of the equivalent east gyroscope.

The sampling data are collected over 3 h as shown in Figure 2, and the sampling frequency is 10 Hz. As shown in Figure 4, after compensation, the bias instability of the equivalent east gyroscope is almost eliminated, but the ARW remains as before. It should be noticed that RRW is almost eliminated through the continuous rotation modulation.

The experiment demonstrated that the RRW and Markov noise could be compensated by continuous rotation alignment, but ARW remained unchanged. The theoretical proofs are shown in Appendix B.

## 5. Experimental Results

**Figure 5:**
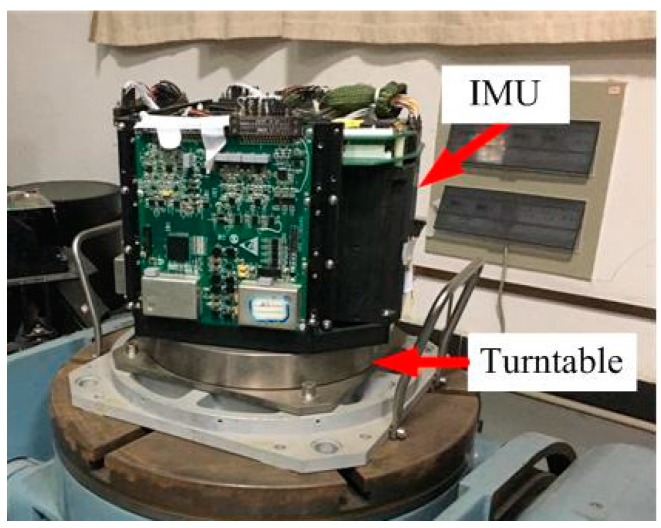
The experimental platform.

The experimental platform is shown in Figure 5.

Considering the installation error, it is difficult to determine the absolute north. The previous north-finding experimental result was used as a reference to evaluate the performance of the approaches. The assumed azimuth was the mean value of 15 experimental results in two weeks north-finding tests. In this study, the experimental north-finding system stayed on a fixed azimuth. For each north-finding algorithm, the north-finding process was repeated five times.

Since the errors of the gyroscopes and accelerometers are unobservable in the fixed-position alignment, which may cause the divergence of the Kalman filter in the practice. We used 5-state Kalman filter for the fixed-position alignment. From Equations (15)–(23), the model can be expressed as follows:

(50) $$\begin{array}{l} x(t)={\left[\delta v_{N},\delta v_{E},\phi_{N},\phi_{E},\phi_{D}\right]}^{T}\\ \dot{x}(t)=F_{1}(t)x(t)+G(t)w(t)\\ w(t)={\left[\begin{array}{ccccc} w_{accx} & w_{accy} & w_{ax} & w_{ay} & w_{az} \end{array}\right]}^{T}\\ z(t)=H_{1}x(t)+\upsilon (t)\;H_{1}=\left[\begin{array}{cc} I_{2\times 2} & 0_{2\times 3} \end{array}\right] \end{array}$$

The coarse alignment method using the gravity in the initial frame as a reference was employed in the experiments [17].

**Figure 6:**
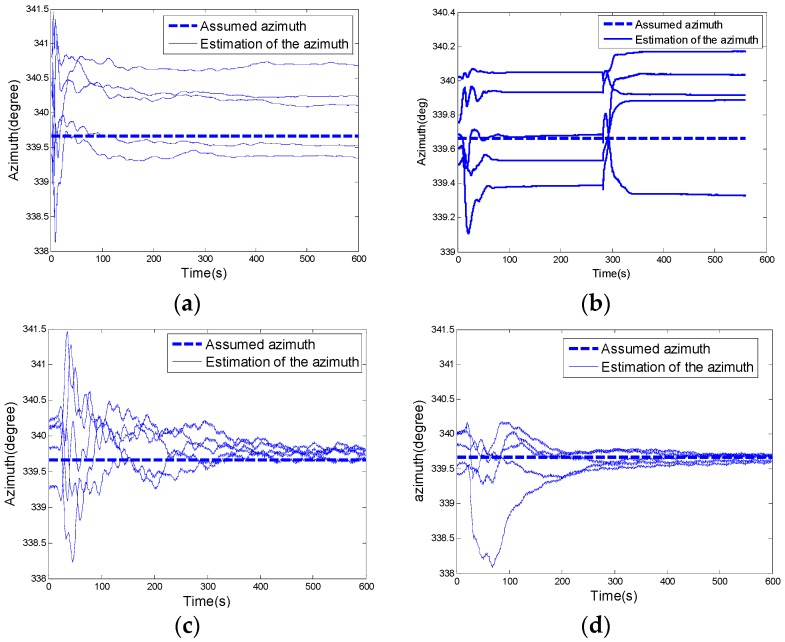
(**a**) The accuracy of the heading angle using the fixed-position alignment; (**b**) The accuracy of the heading angle using the two-position alignment; (**c**) The accuracy of the heading angle using the continuous rotation method; (**d**) The accuracy of the heading angle using the continuous rotation based on the extended observation model.

As shown in Figure 6a–d, the azimuth errors converged with time, the experimental results are coincident with the simulation analysis as shown in Figure 3 in which the new continuous rotation alignment with extended observation is the most efficient algorithm for a Coriolis vibration gyroscope based north-finding system.

In order to further compare the performances of the north-finding methods, we changed the azimuth of the north-finding system to 6 different directions as shown in Equation (51):

(51) $$\varphi_{1}=-20.337{}^{\circ},\;\varphi_{k}=\varphi_{1}+(k-1)60{}^{\circ}\;k=2,\ldots 6$$

**Figure 7:**
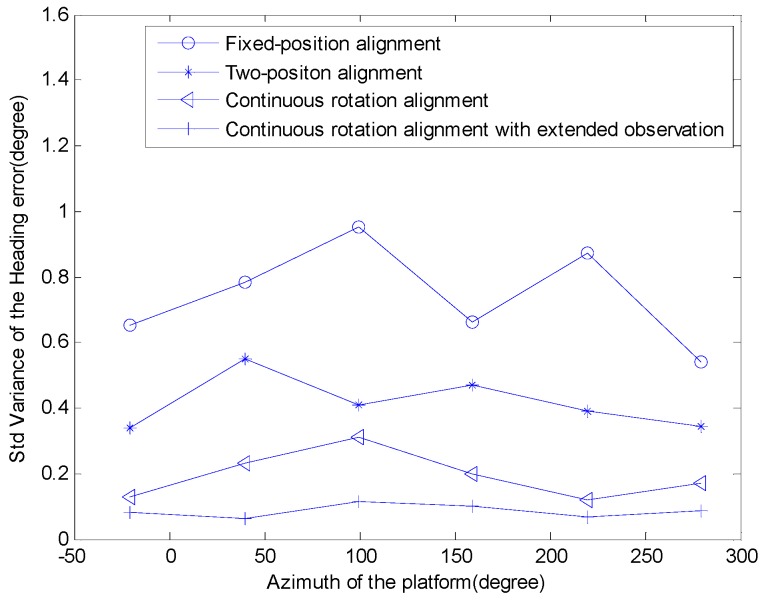
The accuracy of the system using four approaches.

For each azimuth, the north-finding process was repeated for 5 times with the 4 different north-finding algorithms. Then, the RMS of heading errors for each of these algorithms was calculated. As shown in Figure 7, the new approach (continuous rotation alignment with the extended observation model) is the best one, the north-finding accuracy is 0.1° (1*σ*).

## 6. Conclusions

As analyzed in this paper, it is the gyroscope random drift errors that make it a challenge for a cost effective gyroscope based north-finding systems to be achieved. Since it is the equivalent east gyroscope that determines the north-finding accuracy, Allan variance analysis of the equivalent east gyroscope before and after error compensation provides an efficient technique for the evaluation of the gyroscope error estimation.

Comparisons of the Kalman filter convergence rapidity and north-finding accuracy have been made to evaluate the north-finding algorithms. Compared with the other traditional approaches, the new continuous rotation alignment approach based on the extended observation model can improve the north-finding accuracy and convergence rapidity effectively. The experiments have shown that a heading accuracy of 0.1° (1*σ*) can be achieved in 10 min at 28.22° north latitude using a HRG IMU with gyro bias instability of 0.1°/h, compared with 0.6° (1*σ*) north-finding accuracy for the two-position alignment and 1° (1*σ*) for the fixed-position alignment.

In fact, ARW, RRW and Markov noise are the main error source of many gyroscopes (e.g., fiber optic gyroscopes [18]). The new continuous rotation IMU alignment algorithm is not only applicable to the Coriolis vibration gyros (a kind of cost effective HRGs in this paper), but is also suitable for many other gyroscopes with similar stochastic error models.

## Figures and Tables

**Table 1:** Parameters of the error models for the Coriolis vibration gyroscopes (a kind of cost effective HRG in this paper).

Bias Instability $\sigma_{b}$	0.1°/h
ARW $\sigma_{a}$	$0.01{}^{\circ}/\sqrt{h}$
RRW $\sigma_{r}$	$0.3{}^{\circ}/h^{3/2}$
Markov time constant τ	60 s
Markov process driving noise $\sigma_{m}$	$0.02{}^{\circ}/h/\sqrt{s}$

**Table 2:** The azimuth misalignment due to the equivalent east gyroscope errors in 10 min at 28.22° N.

Gyroscope Errors		RMS of Azimuth Misalignment
Bias Instability $\sigma_{bE}$	0.1°/h	$\sigma_{\phi_{Db}}=0.43{}^{\circ}$
ARW $\sigma_{aE}$	$0.01{}^{\circ}/\sqrt{h}$	$\sigma_{\phi_{Da}}=0.10{}^{\circ}$
RRW $\sigma_{rE}$	$0.3{}^{\circ}/h^{3/2}$	$\sigma_{\phi_{Dr}}=0.31{}^{\circ}$
Markov process τ	60 s	$\sigma_{\phi_{Dm}}=0.20{}^{\circ}$
Markov process $\sigma_{mE}$	$0.02{}^{\circ}/h/\sqrt{s}$	

## Appendix A

### Appendix A.1. Proof of Equation (9) (Propagation of the ARW in a North-Finding System) [15,16]

The equivalent east gyroscope integration error $\delta \theta_{aE}$ caused by the ARW can be expressed as follows:

(A1) $$\delta {\dot{\theta }}_{aE}=w_{aE},\;w_{aE}\in N(0,\sigma_{aE}^{2})$$

$P_{aE}$ is the variance of $\delta \theta_{aE}$ which can be expressed as follows:

(A2) $$\begin{array}{l} {\dot{P}}_{aE}=q_{aE}=\sigma_{aE}^{2}\\ P_{aE}(t)=\sigma_{aE}^{2}t \end{array}$$

where $q_{aE}$ is the variance of $w_{aE}$. t is the integration time. Suppose $t_{n}$ is the alignment time, $P_{aE}(t_{n})=\sigma_{aE}^{2}t_{n}$, the RMS of azimuth misalignment $\sigma_{\phi_{Da}}$ due to ARW can be calculated as follows:

(A3) $$\sigma_{\phi_{Da}}=\frac{\sqrt{P_{aE}(t_{n})}}{t_{n}\Omega \cos L}=\frac{\sigma_{aE}\sqrt{t_{n}}}{t_{n}\Omega \cos L}=\frac{\sigma_{aE}}{\sqrt{t_{n}}\Omega \cos L}$$

This completes the proof.

### Appendix A.2. Proof of Equation (10) (Propagation of the RRW in a North-Finding System) [15,16]

The equivalent east gyroscope integration error $\delta \theta_{rE}$ caused by the RRW can be written in matrix form as:

(A4) $$\begin{array}{l} \left[\begin{array}{c} \delta {\dot{\theta }}_{rE}\\ {\dot{\varepsilon }}_{rE} \end{array}\right]=A_{r}\left[\begin{array}{c} \delta \theta_{rE}\\ \varepsilon_{rE} \end{array}\right]+\left[\begin{array}{c} 0\\ w_{rE} \end{array}\right]\\ w_{rE}\in N(0,\sigma_{rE}^{2}) \end{array}$$

The state transition matrix $A_{r}$ and the system noise matrix $q_{r}$ can be written as:

(A5) $$A_{r}=\left[\begin{array}{cc} 0 & 1\\ 0 & 0 \end{array}\right]\;q_{r}=E\{\left[\begin{array}{c} 0\\ w_{rE} \end{array}\right]{\left[\begin{array}{c} 0\\ w_{rE} \end{array}\right]}^{T}\}=\left[\begin{array}{cc} 0 & 0\\ 0 & \sigma_{rE}^{2} \end{array}\right]$$

The state covariance matrix $P_{r}$ can be obtained by calculating the Riccati matrix equation:

(A6) $${\dot{P}}_{r}=A_{r}P_{r}+P_{r}{A_{r}}^{T}+q_{r}$$

(A7) $$P_{r}(t)=\left[\begin{array}{cc} \frac{1}{3}\sigma_{r}^{2}t^{3} & \frac{1}{2}\sigma_{r}^{2}t^{2}\\ \frac{1}{2}\sigma_{r}^{2}t^{2} & \sigma_{r}^{2}t \end{array}\right]$$

(A8) $$\begin{array}{c} P_{rE}(t)=\frac{1}{3}\sigma_{r}^{2}t^{3} \end{array}$$

where $P_{rE}$ is the variance of $\delta \theta_{rE}$. Thus, The RMS of azimuth misalignment $\sigma_{\phi_{Dr}}$ due to the RRW can be calculated as follows:

(A9) $$\sigma_{\phi_{Dr}}=\frac{\sqrt{P_{rE}(t_{n})}}{t_{n}\Omega \cos L}=\frac{\sqrt{\frac{1}{3}\sigma_{rE}^{2}t_{n}^{3}}}{t_{n}\Omega \cos L}=\frac{\sigma_{rE}\sqrt{t_{n}}}{\sqrt{3}\Omega \cos L}$$

This completes the proof.

### Appendix A.3. Proof of Equation (11) (Propagation of the Markov Process in a North-Finding System)

The equivalent east gyroscope integration error $\delta \theta_{mE}$ caused by the Markov process can be expressed in matrix form as follows:

(A10) $$\begin{array}{l} \left[\begin{array}{c} \delta {\dot{\theta }}_{mE}\\ {\dot{\varepsilon }}_{mE} \end{array}\right]=A_{m}\left[\begin{array}{c} \delta \theta_{mE}\\ \varepsilon_{mE} \end{array}\right]+\left[\begin{array}{c} 0\\ w_{mE} \end{array}\right]\\ w_{mE}\in N(0,\sigma_{mE}^{2}) \end{array}$$

The state transition matrix $A_{m}$ and the system noise matrix $q_{m}$ can be written as:

(A11) $$A_{m}=\left[\begin{array}{cc} 0 & 1\\ 0 & -\frac{1}{\tau } \end{array}\right]\;q_{m}=E\{\left[\begin{array}{c} 0\\ w_{mE} \end{array}\right]{\left[\begin{array}{c} 0\\ w_{mE} \end{array}\right]}^{T}\}=\left[\begin{array}{cc} 0 & 0\\ 0 & \sigma_{mE}^{2} \end{array}\right]$$

The state covariance matrix $P_{m}$ can be obtained by calculating the Riccati matrix equation:

(A12) $${\dot{P}}_{m}=A_{m}P_{m}+P_{m}A_{m}{}^{T}+q_{m}$$

(A13) $$\begin{array}{l} P_{m}(t)=\left[\begin{array}{cc} P_{11}(t) & P_{12}(t)\\ P_{21}(t) & P_{22}(t) \end{array}\right]\\ =\left[\begin{array}{cc} \begin{array}{l} \frac{1}{2}\tau^{2}\sigma_{m}^{2}(2t-\tau e^{-\frac{2t}{\tau }}+4\tau e^{-\frac{t}{\tau }}-3\tau )\\ +P_{\varepsilon_{m}}(0)\tau (1-2e^{-\frac{1}{\tau }t}+e^{-\frac{2}{\tau }t}) \end{array} & \frac{1}{2}\tau^{2}\sigma_{m}^{2}{\left(1-e^{-\frac{t}{\tau }}\right)}^{2}+P_{\varepsilon_{m}}(0)\tau (e^{-\frac{1}{\tau }t}-e^{-\frac{2}{\tau }t})\\ \frac{1}{2}\tau^{2}\sigma_{m}^{2}{\left(1-e^{-\frac{t}{\tau }}\right)}^{2}+P_{\varepsilon_{m}}(0)\tau (e^{-\frac{1}{\tau }t}-e^{-\frac{2}{\tau }t}) & \frac{\tau }{2}\sigma_{m}^{2}(1-e^{-\frac{2}{\tau }t})+P_{\varepsilon_{m}}(0)e^{-\frac{2}{\tau }t} \end{array}\right] \end{array}$$

(A14) $$P_{mE}(t)=P_{11}(t)=\frac{1}{2}\tau^{2}\sigma_{m}^{2}(2t-\tau e^{-\frac{2t}{\tau }}+4\tau e^{-\frac{t}{\tau }}-3\tau )+P_{\varepsilon_{m}}(0)\tau (1-2e^{-\frac{1}{\tau }t}+e^{-\frac{2}{\tau }t})$$

$P_{\varepsilon_{mE}}(0)$ is the variance of the initial value of Markov process.

The RMS of azimuth misalignment $\sigma_{\phi_{Dm}}$ due to the Markov process can be calculated as follows:

(A15) $$\sigma_{\phi_{Dm}}=\frac{\sqrt{P_{mE}(t_{n})}}{t_{n}\Omega \cos L}=\frac{\sqrt{\frac{1}{2}\tau^{2}\sigma_{m}^{2}(2t-\tau e^{-\frac{2t}{\tau }}+4\tau e^{-\frac{t}{\tau }}-3\tau )+P_{\varepsilon_{m}}(0)\tau (1-2e^{-\frac{1}{\tau }t}+e^{-\frac{2}{\tau }t})}}{t_{n}\Omega \cos L}$$

This completes the proof.

## Appendix B

### Appendix B.1. Theoretical Proof of the Effects of the Continuous Rotation on the ARW [15]

When the turntable is rotating, the equivalent east gyroscope integration error $\delta \theta_{aE}$ caused by the ARW can be expressed as follows:

(B1) $$\delta {\dot{\theta }}_{aE}=B_{a}\left[\begin{array}{c} w_{ax}\\ w_{ay} \end{array}\right]$$

in which $B_{a}=\left[\begin{array}{cc} \sin\omega_{0}t & \cos\omega_{0}t \end{array}\right]$, $w_{ax}\in N(0,\sigma_{aE}^{2}),w_{ay}\in N(0,\sigma_{aE}^{2})$Suppose:

(B2) $$q_{a}=E\{\left[\begin{array}{c} w_{ax}\\ w_{ay} \end{array}\right]{\left[\begin{array}{c} w_{ax}\\ w_{ay} \end{array}\right]}^{T}\}=\left[\begin{array}{cc} \sigma_{aE}^{2} & 0\\ 0 & \sigma_{aE}^{2} \end{array}\right]$$

The state covariance matrix $P_{a}$ can be calculated as follows:

(B3) $$\begin{array}{c} {\dot{P}}_{a}=B_{a}q_{a}{B_{a}}^{T}=\left[\begin{array}{cc} \sin\omega_{0}t & \cos\omega_{0}t \end{array}\right]q\left[\begin{array}{c} \sin\omega_{0}t\\ \cos\omega_{0}t \end{array}\right]=\sigma_{aE}^{2}\\ P_{a}(t)=\sigma_{aE}^{2}t_{n} \end{array}$$

which is the same as the Equation (A3). Therefore, continuous rotation alignment has no effort on the ARW of the gyroscope.

### Appendix B.2. Theoretical Proof of the Effects of the Continuous Rotation on the RRW [15]

When the turntable is rotating, the equivalent east gyroscope integration error $\delta \theta_{rE}$ caused by the RRW can be expressed as follows:

(B4) $$\begin{array}{l} \left[\begin{array}{c} \delta {\dot{\theta }}_{rE}\\ {\dot{\varepsilon }}_{rx}\\ {\dot{\varepsilon }}_{ry} \end{array}\right]=\left[\begin{array}{ccc} 0 & \sin\omega_{0}t & \cos\omega_{0}t\\ 0 & 0 & 0\\ 0 & 0 & 0 \end{array}\right]\left[\begin{array}{c} \delta \theta_{rE}\\ \varepsilon_{rx}\\ \varepsilon_{ry} \end{array}\right]+\left[\begin{array}{c} 0\\ w_{rx}\\ w_{ry} \end{array}\right]\\ w_{rx}\in N(0,\sigma_{rE}^{2}),w_{ry}\in N(0,\sigma_{rE}^{2}) \end{array}$$

The matrices $A_{r}$, $B_{r}$ and $q_{r}$ are:

(B5) $$\begin{array}{l} A_{r}=\left[\begin{array}{ccc} 0 & \sin\omega_{0}t & \cos\omega_{0}t\\ 0 & 0 & 0\\ 0 & 0 & 0 \end{array}\right]\;B_{r}=I_{2\times 2}\\ q_{r}=E\{\left[\begin{array}{c} 0\\ w_{rx}\\ w_{ry} \end{array}\right]{\left[\begin{array}{c} 0\\ w_{rx}\\ w_{ry} \end{array}\right]}^{T}\}=\left[\begin{array}{ccc} 0 & 0 & 0\\ 0 & \sigma_{rE}^{2} & 0\\ 0 & 0 & \sigma_{rE}^{2} \end{array}\right] \end{array}$$

The state covariance matrix $P_{r}$ can be calculated by Equation (A6), that is:

(B6) $$P_{r}=\left[\begin{array}{ccc} \frac{2\sigma_{rE}^{2}}{\omega_{0}^{2}}(t_{n}-\frac{\sin\omega_{0}t_{n}}{\omega_{0}}) & \begin{array}{l} \frac{\sigma_{rE}^{2}}{\omega_{0}^{2}}(\sin\omega_{0}t_{n}\\ -\omega_{0}t\cos\omega_{0}t_{n}) \end{array} & \begin{array}{l} \frac{\sigma_{rE}^{2}}{\omega_{0}^{2}}(\omega_{0}t_{n}\sin\omega_{0}t_{n}\\ +\cos\omega_{0}t_{n}-1) \end{array}\\ \begin{array}{l} \frac{\sigma_{rE}^{2}}{\omega_{0}^{2}}(\sin\omega_{0}t_{n}\\ -\omega_{0}t_{n}\cos\omega_{0}t_{n}) \end{array} & \sigma_{rE}^{2}t_{n} & 0\\ \begin{array}{l} \frac{\sigma_{rE}^{2}}{\omega_{0}^{2}}(\omega_{0}t_{n}\sin\omega_{0}t_{n}\\ +\cos\omega_{0}t_{n}-1) \end{array} & 0 & \sigma_{rE}^{2}t_{n} \end{array}\right]$$

(B7) $$P_{rE}=\frac{2\sigma_{rE}^{2}}{\omega_{0}^{2}}(t_{n}-\frac{\sin\omega_{0}t_{n}}{\omega_{0}})\approx \frac{2\sigma_{rE}^{2}}{\omega_{0}^{2}}t_{n}$$

Compared with Equations (A2) and(A8), Equation (B7) shows that continuous rotation transforms the RRW into a much small equivalent ARW, which gives an explanation for Figure 5.

When the turntable is rotating, the RMS of azimuth misalignment $\sigma_{\phi_{Dr}}$ due to the RRW can be calculated as follows:

(B8) $$\sigma_{\phi_{Dr}}=\frac{\sqrt{\frac{2\sigma_{rE}^{2}}{\omega_{0}^{2}}(t_{n}-\frac{\sin\omega_{0}t_{n}}{\omega_{0}})}}{t_{n}\Omega \cos L}=\frac{\sigma_{rE}\sqrt{2(t_{n}-\frac{\sin\omega_{0}t_{n}}{\omega_{0}})}}{\omega_{0}t_{n}\Omega \cos L}$$

Assuming the alignment time $t_{n}$ is 10 min, the rotation rate of the turntable is 10°/s, the variance of the RRW is $0.02{}^{\circ}/h^{3/2}$, the RMS values $\sigma_{\phi_{Dr}}$ of the azimuth misalignment due to the RRW can be obtained based on Equations (A9) and (B8).

When the turntable is not rotating:

(B9) $$\sigma_{\phi_{Dr}}=\frac{\sigma_{rE}\sqrt{t_{n}}}{\sqrt{3}\Omega \cos L}=0.020({}^{\circ})$$

When the turntable is rotating:

(B10) $$\sigma_{\phi_{Dr}}=\frac{\sigma_{rE}\sqrt{2(t_{n}-\frac{\sin\omega_{0}t_{n}}{\omega_{0}})}}{\omega_{0}t_{n}\Omega \cos L}=4.8\times 10^{-4}({}^{\circ})$$

Thus, the RRW of the gyroscope can be eliminated by continuous rotation alignment.

### Appendix B.3. Theoretical Proof of the Effects of the Continuous Rotation on the Markov Noise

When the turntable is rotating, the equivalent east gyroscope integration error $\delta \theta_{mE}$ caused by the Markov noise can be expressed as follows:

(B11) $$\begin{array}{l} \left[\begin{array}{c} \delta {\dot{\theta }}_{mE}\\ {\dot{\varepsilon }}_{mx}\\ {\dot{\varepsilon }}_{my} \end{array}\right]=\left[\begin{array}{ccc} 0 & \sin\omega_{0}t & \cos\omega_{0}t\\ 0 & -\frac{1}{\tau } & 0\\ 0 & 0 & -\frac{1}{\tau } \end{array}\right]\left[\begin{array}{c} \delta \theta_{mE}\\ \varepsilon_{mx}\\ \varepsilon_{my} \end{array}\right]+\left[\begin{array}{c} 0\\ w_{mx}\\ w_{my} \end{array}\right]\\ w_{mx}\in N(0,\sigma_{m}^{2}),w_{my}\in N(0,\sigma_{m}^{2}) \end{array}$$

The matrix $A_{m}$, $B_{m}$ and $q_{m}$ is:

(B12) $$\begin{array}{cc} A_{m}=\left[\begin{array}{ccc} 0 & \sin\omega_{0}t & \cos\omega_{0}t\\ 0 & -\frac{1}{\tau } & 0\\ 0 & 0 & -\frac{1}{\tau } \end{array}\right]\;B_{m}=I_{3\times 3} & q_{m}=E\{\left[\begin{array}{c} 0\\ w_{mx}\\ w_{my} \end{array}\right]{\left[\begin{array}{c} 0\\ w_{mx}\\ w_{my} \end{array}\right]}^{T}\}=\left[\begin{array}{ccc} 0 & 0 & 0\\ 0 & \sigma_{mE}^{2} & 0\\ 0 & 0 & \sigma_{mE}^{2} \end{array}\right] \end{array}$$

The state covariance matrix $P_{m}$ can be calculated by Equation (A3), that is:

(B13) $$P_{m}=\left[\begin{array}{ccc} \begin{array}{l} \frac{\tau^{2}\sigma_{m}^{2}}{2{\left(1+\tau^{2}{\omega_{0}}^{2}\right)}^{2}}(-\tau e^{-\frac{2t}{\tau }}-3\tau +2t+\tau^{3}{\omega_{0}}^{2}(1-e^{-\frac{2t}{\tau }})\\ +2\tau^{2}{\omega_{0}}^{2}t+4\tau \cos\omega_{0}te^{-\frac{t}{\tau }}-4\tau^{2}\omega_{0}\sin\omega_{0}te^{-\frac{t}{\tau }})\\ +\frac{\tau^{2}P_{\varepsilon_{mE}}(0)}{{\left(1+\tau^{2}{\omega_{0}}^{2}\right)}^{2}}(1+e^{-\frac{2t}{\tau }}+\tau^{2}{\omega_{0}}^{2}(1+e^{-\frac{2t}{\tau }})\\ -2e^{-\frac{t}{\tau }}\cos\omega_{0}t-2\tau^{2}{\omega_{0}}^{2}e^{-\frac{t}{\tau }}\cos\omega_{0}t) \end{array} & * & *\\ \begin{array}{l} -\frac{\tau^{2}\sigma_{m}^{2}}{2\left(1+\tau^{2}{\omega_{0}}^{2}\right)}(-\tau \omega_{0}e^{-\frac{2t}{\tau }}\cos\omega_{0}t+\tau \omega_{0}\cos\omega_{0}t\\ -e^{-\frac{2t}{\tau }}\sin\omega_{0}t-\sin\omega_{0}t)-\frac{\tau P_{\varepsilon_{mE}}(0)}{1+a^{2}{\omega_{0}}^{2}}(-\tau \omega_{0}e^{-\frac{t}{a}}\\ +e^{-\frac{2t}{a}}\sin\omega_{0}t+\tau \omega_{0}e^{-\frac{2t}{a}}\cos\omega_{0}t) \end{array} & \begin{array}{l} \frac{\tau }{2}\sigma_{m}^{2}(1-e^{-\frac{2}{\tau }t})\\ +P_{\varepsilon_{mE}}(0)e^{-\frac{2}{\tau }t} \end{array} & *\\ \begin{array}{l} \frac{\tau^{2}\sigma_{m}^{2}}{2\left(1+\tau^{2}{\omega_{0}}^{2}\right)}(-2e^{-\frac{t}{\tau }}+e^{-\frac{2t}{\tau }}\cos\omega_{0}t\\ +\cos\omega_{0}t-\tau \omega_{0}e^{-\frac{2t}{\tau }}\sin\omega_{0}t+\tau \omega_{0}\sin\omega_{0}t)\\ +\frac{\tau P_{\varepsilon_{mE}}(0)}{1+\tau^{2}{\omega_{0}}^{2}}(e^{-\frac{t}{\tau }}-e^{-\frac{2t}{\tau }}\cos\omega_{0}t+\tau \omega_{0}e^{-\frac{2t}{\tau }}\sin\omega_{0}t) \end{array} & 0 & \begin{array}{l} \frac{\tau }{2}\sigma_{m}^{2}(1-e^{-\frac{2}{\tau }t})\\ +P_{\varepsilon_{mE}}(0)e^{-\frac{2}{\tau }t} \end{array} \end{array}\right]$$

(B14) $$\begin{array}{ll} P_{mE} & =\frac{\tau^{2}\sigma_{m}^{2}}{2{\left(1+\tau^{2}{\omega_{0}}^{2}\right)}^{2}}[-\tau e^{-\frac{2t}{\tau }}-3\tau +2t+\tau^{3}{\omega_{0}}^{2}(1-e^{-\frac{2t}{\tau }})+2\tau^{2}{\omega_{0}}^{2}t+4\tau \cos\omega_{0}te^{-\frac{t}{\tau }}-4\tau^{2}\omega_{0}\sin\omega_{0}te^{-\frac{t}{\tau }}]\\ & +\frac{\tau^{2}P_{\varepsilon_{mE}}(0)}{{\left(1+\tau^{2}{\omega_{0}}^{2}\right)}^{2}}[1+e^{-\frac{2t}{\tau }}+\tau^{2}{\omega_{0}}^{2}(1+e^{-\frac{2t}{\tau }})-2e^{-\frac{t}{\tau }}\cos\omega_{0}t-2\tau^{2}{\omega_{0}}^{2}e^{-\frac{t}{\tau }}\cos\omega_{0}t]\\ & \approx \frac{\tau^{2}\sigma_{m}^{2}}{2{\left(1+\tau^{2}{\omega_{0}}^{2}\right)}^{2}}[-\tau e^{-\frac{2t}{\tau }}-3\tau +2t+\tau^{3}{\omega_{0}}^{2}(1-e^{-\frac{2t}{\tau }})+2\tau^{2}{\omega_{0}}^{2}t]+\frac{\tau^{2}P_{\varepsilon_{mE}}(0)}{{\left(1+\tau^{2}{\omega_{0}}^{2}\right)}^{2}}[1+e^{-\frac{2t}{\tau }}+\tau^{2}{\omega_{0}}^{2}(1+e^{-\frac{2t}{\tau }})] \end{array}$$

When the turntable is rotating, the RMS of azimuth misalignment $\sigma_{\phi_{Dm}}$ due to the RRW can be calculated as follows:

(B15) $$\begin{array}{cc} \sigma_{\phi_{Dm}} & =\frac{\sqrt{P_{mE}(t_{n})}}{t_{n}\Omega \cos L}\\ & =\frac{\tau \sqrt{\frac{1}{2}\sigma_{m}^{2}(-\tau e^{-\frac{2t}{\tau }}-3\tau +2t+\tau^{3}{\omega_{0}}^{2}(1-e^{-\frac{2t}{\tau }})+2\tau^{2}{\omega_{0}}^{2}t)+P_{\varepsilon_{mE}}(0)(1+e^{-\frac{2t}{\tau }}+\tau^{2}{\omega_{0}}^{2}(1{+e}^{-\frac{2t}{\tau }}))}}{t_{n}\Omega \cos L(1+\tau^{2}{\omega_{0}}^{2})} \end{array}$$

Similar to the theoretical proof of the RRW, assuming the alignment time $t_{n}$ is 10 min, the rotation rate of the turntable is 10°/s, the Markov time constant is 60 s, the variance of the Markov driving noise is $0.02{}^{\circ}/h/\sqrt{s}$, the RMS values $\sigma_{\phi_{Dm}}$ of the azimuth misalignment due to the Markov noise can be obtained based on Equations (A15) and (B15).

When the turntable is not rotating:

(B16) $$\sigma_{\phi_{Dm}}=\frac{\sqrt{\frac{1}{2}\tau^{2}\sigma_{m}^{2}(2t-\tau e^{-\frac{2t}{\tau }}+4\tau e^{-\frac{t}{\tau }}-3\tau )+P_{\varepsilon_{mE}}(0)\tau (1-2e^{-\frac{1}{\tau }t}+e^{-\frac{2}{\tau }t})}}{t_{n}\Omega \cos L}=0.21({}^{\circ})$$

When the turntable is rotating:

(B17) $$\begin{array}{cc} \sigma_{\phi_{Dm}} & =\frac{\tau \sqrt{\frac{1}{2}\sigma_{m}^{2}(-\tau e^{-\frac{2t}{\tau }}-3\tau +2t+\tau^{3}{\omega_{0}}^{2}(1-e^{-\frac{2t}{\tau }})+2\tau^{2}{\omega_{0}}^{2}t)+P_{\varepsilon_{mE}}(0)(1+e^{-\frac{2t}{\tau }}+\tau^{2}{\omega_{0}}^{2}(1{+e}^{-\frac{2t}{\tau }}))}}{t_{n}\Omega \cos L(1+\tau^{2}{\omega_{0}}^{2})}\\ & =0.02({}^{\circ}) \end{array}$$

Thus, the Markov noise of the gyroscope can be eliminated by continuous rotation alignment.
